# Deep Brain Stimulation Is Effective for Treatment-Resistant Depression: A Meta-Analysis and Meta-Regression

**DOI:** 10.3390/jcm9092796

**Published:** 2020-08-30

**Authors:** Frederick L. Hitti, Andrew I. Yang, Mario A. Cristancho, Gordon H. Baltuch

**Affiliations:** 1 Department of Neurosurgery, Pennsylvania Hospital, University of Pennsylvania, 800 Spruce St, Philadelphia, PA 19107, USA; andrew.yang@pennmedicine.upenn.edu (A.I.Y.); gordon.baltuch@pennmedicine.upenn.edu (G.H.B.); 2 Department of Psychiatry, University of Pennsylvania, 3535 Market Street, Philadelphia, PA 19104, USA; marioc@pennmedicine.upenn.edu

**Keywords:** deep brain stimulation, treatment-resistant depression, depression, meta-analysis, meta-regression, subcallosal cingulate gyrus, medial forebrain bundle, inferior thalamic peduncle, ventral capsule, ventral striatum

## Abstract

Major depressive disorder (MDD) is a leading cause of disability and a significant cause of mortality worldwide. Approximately 30–40% of patients fail to achieve clinical remission with available pharmacological treatments, a clinical course termed treatment-resistant depression (TRD). Numerous studies have investigated deep brain stimulation (DBS) as a therapy for TRD. We performed a meta-analysis to determine efficacy and a meta-regression to compare stimulation targets. We identified and screened 1397 studies. We included 125 citations in the qualitative review and considered 26 for quantitative analysis. Only blinded studies that compared active DBS to sham stimulation (k = 12) were included in the meta-analysis. The random-effects model supported the efficacy of DBS for TRD (standardized mean difference = −0.75, <0 favors active stimulation; *p* = 0.0001). The meta-regression did not demonstrate a statistically significant difference between stimulation targets (*p* = 0.45). While enthusiasm for DBS treatment of TRD has been tempered by recent randomized trials, this meta-analysis reveals a significant effect of DBS for the treatment of TRD. Additionally, the majority of trials have demonstrated the safety and efficacy of DBS for this indication. Further trials are required to determine the optimal stimulation parameters and patient populations for which DBS would be effective. Particular attention to factors including electrode placement technique, patient selection, and long-term follow-up is essential for future trial design.

## 1. Introduction

Major depressive disorder (MDD) is one of the most common psychiatric diseases, and while a number of therapies are available, many patients remain symptomatic despite treatment [1,2]. Well-established treatment modalities for MDD include psychotherapy, medication, and electroconvulsive therapy (ECT) [3,4,5,6,7,8,9]. While ECT is efficacious for many patients resistant to medication and therapy, there are significant adverse effects associated with ECT, including cognitive and memory dysfunction [3]. Furthermore, there are patients who are refractory to multiple available therapies, including ECT. Patients who fail to improve following treatment with two or more therapies are considered to have treatment-resistant depression (TRD) [5,8,10,11,12]. Due to the considerable number of treatment non-remitters (30–40% of patients with MDD), developing novel therapies for TRD represents a major unmet need.

Deep brain stimulation (DBS) is a technique that uses implanted intracranial electrodes to modulate neural activity. It is currently a well-established, FDA-approved treatment for movement disorders such as Parkinson’s disease (PD) and essential tremor (ET) [13,14]. In addition to movement disorders, DBS has been explored as a treatment modality for psychiatric conditions. Multiple human trials have explored the efficacy of DBS for TRD. Anatomic targets have included the ventral anterior limb of the internal capsule (vALIC) [15], ventral capsule/ventral striatum (VC/VS) [16], subcallosal cingulate (SCC) [17,18,19,20,21,22], inferior thalamic peduncle (ITP) [23], medial forebrain bundle (MFB) [24,25], and lateral habenula [26]. Reports regarding the efficacy of DBS for TRD have been mixed, with some studies demonstrating encouraging results, while others have shown a lack of efficacy relative to sham stimulation. To leverage all of the available data, we performed a meta-analysis to determine the efficacy of DBS for TRD. We then performed a meta-regression to compare stimulation targets. While prior meta-analyses have been undertaken [27,28,29], here we included only studies that compared active to sham stimulation in a blinded fashion. Furthermore, our analysis includes more recent studies.

## 2. Methods

### 2.1. Search Strategy

We used the PubMed database to identify studies investigating DBS for MDD and screened all studies for inclusion. We used the following search terms to identify relevant studies: (“deep brain stimulation”[MeSH Terms] OR (“deep”[All Fields] AND “brain”[All Fields] AND “stimulation”[All Fields]) OR “deep brain stimulation”[All Fields] OR “DBS”[All Fields]) AND (“depressive disorder”[MeSH Terms] OR (“depressive”[All Fields] AND “disorder”[All Fields]) OR “depressive disorder”[All Fields] OR “depression”[All Fields] OR “depression”[MeSH Terms]). All studies were considered, including studies written in other languages. The search was conducted on 10/16/2019, and the analysis followed the Preferred Reporting Items for Systematic reviews and Meta-Analyses (PRISMA) guidelines.

### 2.2. Study Inclusion and Exclusion Criteria

Only studies that investigated the efficacy of DBS for MDD were included. We excluded studies that utilized other therapies to treat MDD (e.g., ECT, epidural stimulation, vagal nerve stimulation, transcranial magnetic stimulation, or tDCS). We also excluded studies that investigated comorbid depression in the context of other disorders, such as epilepsy, dystonia, Tourette syndrome, anorexia nervosa, obsessive–compulsive disorder, schizophrenia, headache, ET, and PD. We excluded all non-human studies. Of the studies relevant to DBS as a treatment for MDD, we excluded case reports, non-systematic reviews, perspectives, commentaries, editorials, and opinions. We included the remainder of the studies for our qualitative review. For the quantitative meta-analysis, we only included studies in which sham stimulation was compared to active stimulation in a blinded fashion (either single- or double-blind). The clinical trial designs were varied and included both crossover and parallel studies.

### 2.3. Data Extraction and Outcome Measures

Our primary outcome was the efficacy of DBS as a treatment for depression as assessed by changes in the Hamilton Depression Rating Scale (HDRS) or Montgomery–Åsberg Depression Rating Scale (MADRS) scores. We compared sham stimulation scores to active stimulation scores. The data were extracted from tables when provided. If tables with the raw data were not provided, the WebPlotDigitizer tool was used to extract data from published graphs. We also extracted the number of patients, stimulation target, side effects of treatment, adverse events, study design, and depression rating scale used.

### 2.4. Statistical Methods

Statistical analysis was conducted in R using the meta, metaphor, and dmetar packages. We followed the guide published by Harrer et al. to conduct the analysis [30]. A random-effects model was employed for the meta-analysis to account for differences in study populations. We used the DerSimonian–Laird estimator for τ^2^ (variance of true effect magnitude distributions), as it is the most widely used estimator. The studies included in the quantitative analysis used different depression rating scales. Therefore, we computed standardized mean differences so that the studies could be compared. We also calculated heterogeneity (I^2^) of the studies in R. The differential efficacies of the various stimulation targets were compared with mixed-effects meta-regression. R was used to generate funnel plots and conduct Egger’s test. Means are presented with their corresponding standard deviations. A *p*-value < 0.05 was considered statistically significant.

## 3. Results

**Figure 1:**
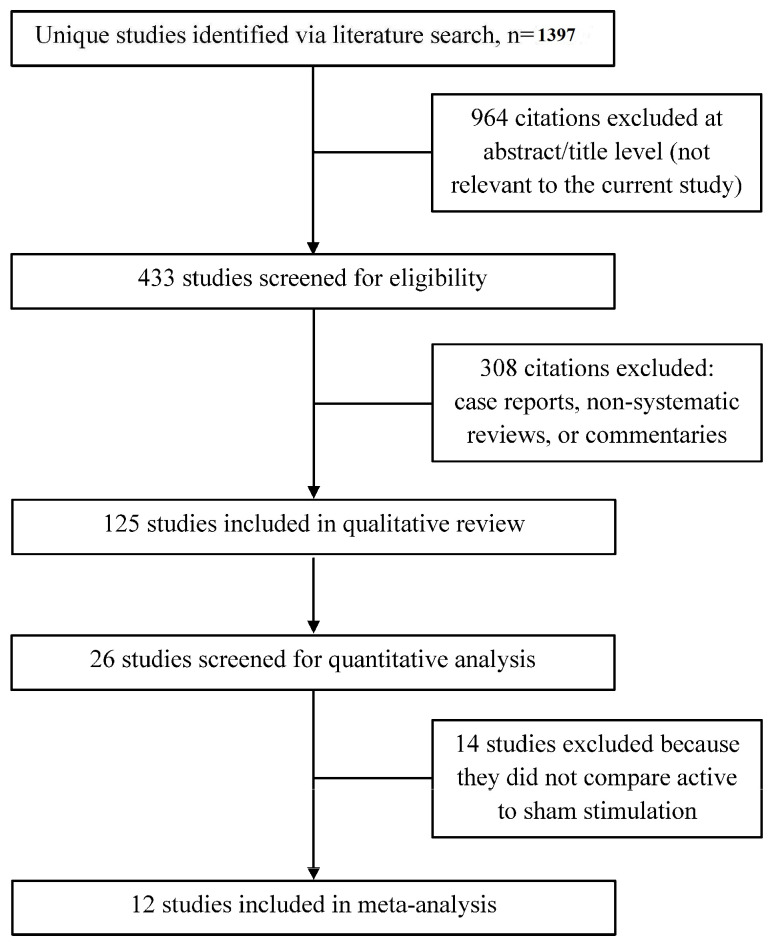
Flowchart of studies selected for inclusion in the qualitative review and quantitative meta-analysis.

We used fairly broad search terms (see Section 2) to ensure the inclusion of all studies relevant to the use of DBS as a therapy for depression. Our search identified 1397 studies, and all were screened for inclusion (Figure 1). We excluded 964 studies at the abstract/title level because these studies either did not use DBS as the therapeutic modality, examined depressive symptomatology in the context of other diseases, or were non-human animal studies. Of the remaining relevant studies, 308 were excluded because they were case reports, non-systematic reviews, perspectives, commentaries, editorials, or opinions. The remaining 125 studies were included in our qualitative review. We then screened these studies for inclusion in our quantitative meta-analysis. Twenty-six studies were candidates for inclusion at the abstract level. Thirteen studies were excluded because they did not compare active to sham stimulation [31,32,33,34,35,36,37,38,39,40,41,42,43], and one study was excluded as it included only three patients [44]. Therefore, 12 studies [15,16,17,18,19,20,21,22,23,24,25] (186 unique patients) were included in the meta-analysis and meta-regression (Table 1). The Raymaekers et al. study was analyzed as two separate studies, because this study included two anatomically distinct stimulation targets, and both targets were evaluated with blinded stimulation periods.

The studies included in the meta-analysis had varied trial designs (Table 1). Due to our inclusion criteria, all studies contained a period of blinded sham stimulation and blinded active stimulation. The duration of the active and sham stimulation periods, however, was heterogeneous. The average blinded stimulation duration was 7.5 ± 6.6 weeks. All studies contained an open-label period of long-term active stimulation following the blinded phases. These long-term data were not included in the meta-analysis, since the goal of the present study was to compare blinded active stimulation to blinded sham stimulation. The majority of the trials (75%) were done in a crossover fashion (Table 1). Thus, all of the patients in these studies received both active and sham stimulation in a blinded fashion. Importantly, these study designs allow for within-subject comparisons and may enhance statistical power.

**Figure 2:**
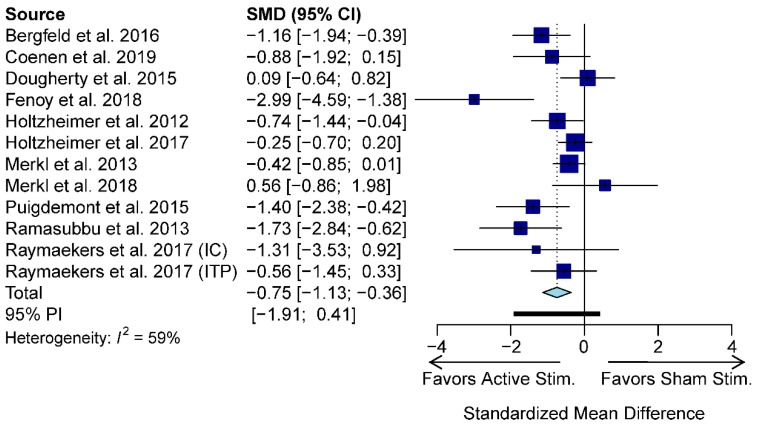
Meta-analysis forest plot depicting changes in HDRS/MADRS scores with active stimulation compared to sham stimulation. CI: confidence interval; IC: internal capsule; ITP: inferior thalamic peduncle; SMD: standardized mean difference; PI: prediction interval.

Using a random-effects model, our meta-analysis revealed that active stimulation results in a greater decline in HDRS/MADRS scores relative to sham stimulation (standardized mean difference (SMD) = −0.75; −1.13 to −0.36, 95% confidence interval (CI); *p*-value = 0.0001; Figure 2). There was moderate heterogeneity across studies (I^2^ = 59%).

**Figure 3:**
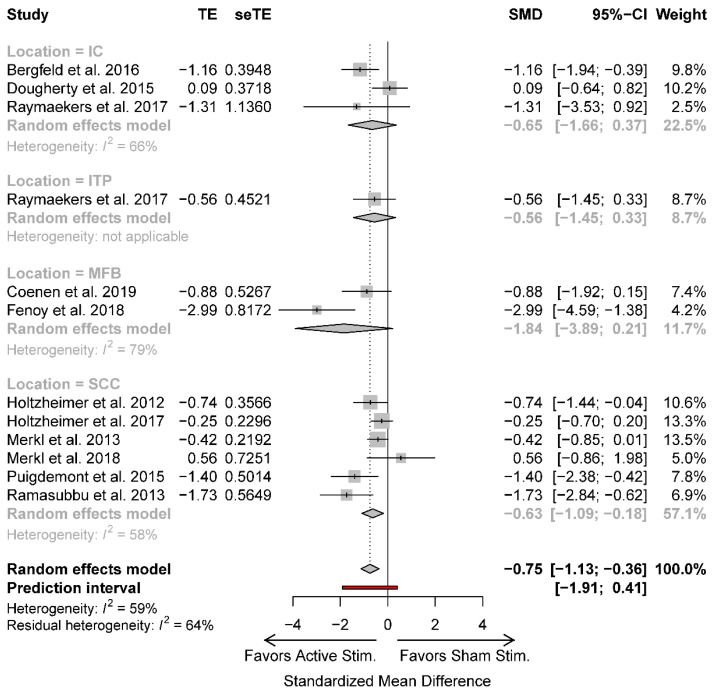
Meta-regression forest plot comparing various stimulation targets. CI: confidence interval; IC: internal capsule; ITP: inferior thalamic peduncle; MFB: medial forebrain bundle; SCC: subcallosal cingulate; SMD: standardized mean difference; TE: treatment effect; seTE: standard error of treatment effect.

In addition to differences in study design, the studies also investigated the efficacy of DBS for TRD using different stimulation targets (Table 1). The most common target was the SCC (50% of studies), followed by the internal capsule (IC, 25%), MFB (17%), and ITP (8%). While there were a limited number of studies, we utilized meta-regression to determine if the available data would reveal an optimal stimulation target. The meta-regression, however, did not demonstrate a statistically significant difference (*p* = 0.45) between stimulation targets (Figure 3).

Since the duration of stimulation during the blinded phase varied between studies, we performed another meta-regression to determine if there was an association between the duration of active stimulation and SMD. Our analysis did not reveal a significant effect of stimulation duration on SMD outcomes (*p* = 0.20).

**Figure 4:**
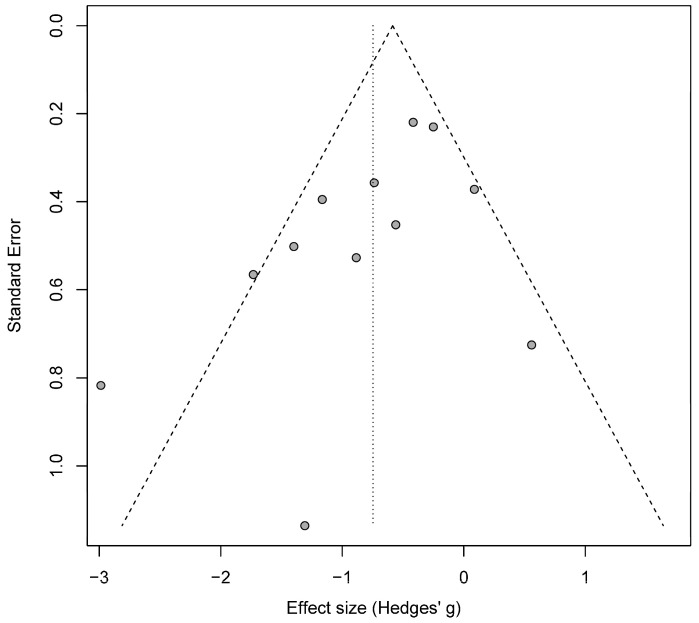
Funnel plot of studies included in the quantitative analysis.

Publication bias is an important concern when conducting a meta-analysis. We investigated for possible publication bias by first generating a funnel plot (Figure 4). We then tested for asymmetry of the funnel plot with Egger’s test. The test revealed that there was no statistically significant asymmetry in the plot (intercept −1.9; 95% CI −3.864–0.056; *p* = 0.07), thus arguing against publication bias. Given the strong trend of Egger’s test and the fact that one study (Fenoy et al. 2018) was a clear outlier, as depicted in the funnel plot, we re-analyzed the data with this outlier study excluded. Using a random-effects model, a meta-analysis of the pared data confirmed that active stimulation results in a greater decline in HDRS/MADRS scores relative to sham stimulation (SMD = −0.62; 95% CI −0.95 to −0.30; *p* = 0.0002). Removing the outlier study decreased study heterogeneity (I^2^ = 45%) and decreased the likelihood of publication bias as estimated by Egger’s test (intercept −1.3; 95% CI −3.26–0.66; *p* = 0.21).

We examined and compiled the adverse events reported in the studies included in the quantitative meta-analysis. The adverse events occurring in greater than 1% of patients are listed in Table 2, and the full list of adverse events in each study is detailed in Supplementary Table S1. The most common complaint was headache (26% of patients), followed by visual disturbances (21%), worsening depression (16%), sleep disturbances (16%), and anxiety (14%). All other adverse events were only seen in less than 10% of patients (Table 2). The authors of the original studies reported that the vast majority of adverse events were transient and were often resolved by stimulation parameter adjustment. The headaches were often postoperative and resolved a few days after surgery. A significant number of patients (*n* = 16, 8%) expressed suicidal ideation, and a similar number of patients (*n* = 15, 8%) attempted suicide. Completed suicides were rare. In two studies, one patient from each study who had no response to DBS committed suicide [15,16]. In one large study, there were two deaths by suicide in the control group during the open-label phase [17]. Finally, in another study, two patients committed suicide [23]. These suicides were deemed to be unrelated to DBS, because both patients had a history of suicide attempts and DBS did not appear to increase impulsivity [23].

## 4. Discussion

MDD is a very prevalent neuropsychiatric condition. Although there are a number of existing treatment modalities, many patients remain symptomatic despite adequate treatment protocols. There is an urgent need for additional treatment options for TRD, since it is a significant source of morbidity and mortality.

Pathological neural activity (either hyperactivity or hypoactivity) may lead to neurological or psychiatric disease, and non-pharmacological neuromodulatory techniques may be used to ameliorate these conditions. DBS is a neuromodulatory approach that is an FDA-approved treatment for movement disorders, so multiple investigators have trialed DBS as a therapy for TRD. Initial open-label reports regarding the efficacy of DBS for TRD were encouraging [36,45], and subsequently, multiple randomized trials were initiated and completed [16,17]. The large-scale trials, however, did not reveal a significant difference between the control and treatment groups. A number of reasons have been posited to explain these negative results, including choice of stimulation target, electrode placement technique, patient selection, and short-term follow-up of patients [43,46,47,48,49,50,51,52].

Due to the discrepancy between earlier reports and larger trials, we undertook a meta-analysis to investigate the efficacy of DBS for TRD. In agreement with prior analyses [27,28,29], we found a significant effect of DBS on TRD. Specifically, active stimulation was associated with an improvement in depression scores relative to sham stimulation. The effect size of this treatment is medium to large, as indicated by the SMD of −0.75 [53]. This effect size is much larger than the small effect sizes (~0.3) seen with pharmacologic antidepressant treatment of patients with MDD [54]. Our findings extend those of prior analyses by including all currently available studies and by only including blinded studies that utilized a trial design in which active stimulation was compared to sham stimulation. Importantly, analyzing the data in this manner allows for the control of placebo effects. While some patients are able to detect active stimulation due to side effects such as visual disturbances, these effects are largely absent with a stimulation parameter adjustment [24,25]. With most studies including an optimization phase prior to the blinded assessments, it is reasonable to propose that comparing active stimulation to sham stimulation controls for placebo effects.

We conducted a meta-regression to determine if the data would reveal an optimal stimulation target. Due to the limited number of available studies, our analysis did not demonstrate an optimal target. With further research, the answer to this question may be elucidated in the future; however, a significant limitation is the heterogeneous nature of MDD neurobiology (i.e., symptoms of depression as a common manifestation of multiple brain functional abnormalities). It may be that instead of one optimal stimulation target for all patients, the optimal stimulation target varies across individuals [46,55,56,57,58].

As with any therapy, there is a risk of publication bias since studies with positive results and large effect sizes are more likely to be published than studies with small effect sizes or negative results [59]. To investigate publication bias in DBS for depression, we plotted the studies in a funnel plot and used Egger’s test to assess for asymmetry. We did not find evidence of publication bias in this series of studies.

DBS is a well-tolerated treatment for movement disorders, but it is important to critically evaluate potential side effects of DBS for depression. The thoroughness of adverse event reporting varied between the studies. Nonetheless, the majority of adverse events were transient. Furthermore, many side effects were relieved by a stimulation parameter adjustment, as is seen with DBS for movement disorders. Patients with MDD are at significant risk for suicide, and patients with TRD have an even higher risk of suicide [60,61]. Currently available data do not demonstrate an increased risk of suicide with DBS, since suicide was rare in these studies and occurred at a lower rate than in patients with severe MDD not receiving DBS [62]. Moreover, the completed suicides occurred in non-responders or were deemed unrelated to DBS. As an invasive therapy, DBS may be perceived by patients as a “last resort” for recovery. Therefore, non-responders may represent a particularly high-risk group for suicide. Psychoeducation and adequate discussion of post DBS treatment options to reframe those perceptions may decrease the risk of suicide. In summary, published trials have demonstrated the safety of DBS for TRD.

While the results of this meta-analysis are encouraging, additional large-scale clinical trials demonstrating the efficacy of DBS for TRD are essential. Crucial to these efforts will be careful consideration of future trial design [24,63]. The large-scale clinical trials of DBS for TRD conducted to date have utilized a parallel trial design, whereas many of the smaller trials employed a crossover design (Table 1). Given the negative outcomes of the large-scale trials, a crossover design may be optimal to investigate the efficacy of DBS for TRD. As a therapy, DBS is unique in that sham stimulation and active stimulation may be compared within individuals, thereby facilitating crossover trial design. Crossover studies require fewer patients to achieve significance, so this design optimizes statistical power [64]. Some studies have used customized trial designs with a variable optimization period [15,65]. Ensuring proper optimization prior to randomization may be necessary to determine the efficacy of DBS for TRD. Another important factor in trial design is the length of time that the patient undergoes therapy. Long-term open-label studies have demonstrated that the efficacy of DBS for TRD improves over time, so longer trials may be necessary [17,18,38,66,67]. Finally, depression severity waxes and wanes throughout a patient’s disease course. Therefore, trial designs that compare groups at specific time points may not be optimal. Integrating scores over predefined time periods may enhance outcome assessment.

In addition to trial design, proper electrode targeting is important for the efficacy of DBS for the treatment of depression. Instead of targeting brain nuclei, as is routinely done, targeting fiber tracts using patient-specific tractography may be important for therapeutic efficacy [49,50,51,52,58,68]. Furthermore, stereotactic accuracy of electrode placement is essential. For example, intraoperative phenomena, such as brain shift, should be accounted for [48]. Patient selection is also a critical consideration for future studies. The available data have demonstrated that there is a subgroup of patients that respond to this therapy and a subgroup that does not. Determining patient-specific factors (e.g., anatomical or symptom-based) that predict response to DBS for TRD may enable targeted selection of patients for whom DBS would be therapeutic [43,69,70,71,72,73,74,75]. These patient-specific factors could then be used as study inclusion criteria to enhance the probability of study success.

## 5. Conclusions

While enthusiasm for DBS as a therapy for TRD has been tempered by recent randomized trials, this meta-analysis reveals that active stimulation significantly ameliorates depression in patients with TRD. The meta-regression did not reveal an optimal stimulation target to treat depression due to the small size and number of available studies, and also likely due to the heterogeneous nature of this condition’s neurobiology. Additional trials are needed to determine optimal stimulation targets. Further studies are also required to establish the patient populations for whom DBS would be effective. Particular attention to factors that include electrode placement technique, patient selection, and long-term observation is essential for future trial design.

## Figures and Tables

**Table 1:** Studies included in meta-analysis and meta-regression.

Study	Location	N	Blinded Crossover
Bergfeld et al. 2016	vALIC	16	Yes
Coenen et al. 2019	MFB	16	No
Dougherty et al. 2015	VC/VS	29	No
Fenoy et al. 2018	MFB	6	Yes
Holtzheimer et al. 2012	SCC	10	Yes
Holtzheimer et al. 2017	SCC	85	No
Merkl et al. 2013	SCC	6	Yes
Merkl et al. 2018	SCC	4	Yes *
Puigdemont et al. 2015	SCC	5	Yes
Ramasubbu et al. 2013	SCC	4	Yes
Raymaekers et al. 2017	IC/BST	5	Yes
Raymaekers et al. 2017	ITP	5	Yes

* Only half of the patients crossed over. IC/BST: internal capsule/bed nucleus of the stria terminalis; ITP: inferior thalamic peduncle; MFB: medial forebrain bundle; SCC: subcallosal cingulate; vALIC: ventral anterior limb of the internal capsule; VC/VS: ventral capsule/ventral striatum.

**Table 2:** Adverse events.

Adverse Event	Patients (N)	Patients (%)
Headache	50	26
Blurred Vision/Diplopia	41	21
Worsening depression	31	16
Sleep Disturbances	30	16
Anxiety	26	14
Pain Around Neurostimulator	17	9
Nausea	16	8
Suicidal Ideation	16	8
Pain Around Incisions	16	8
Post-operative discomfort	16	8
Suicide Attempt	15	8
Device Infection	15	8
Balance/Gait Problems	13	7
Non-Specific Somatic Complaints	12	6
Pain/pulling sensation around Extension Wires	11	6
Other infections	10	5
Agitation	9	5
Paresthesias	9	5
Restlessness	8	4
Disinhibition/Impulsivity	8	4
Hypomania	8	4
Confusion/Cognitive impairment	8	4
Swollen Eyes	6	3
Excessive Sweating	6	3
Memory Disturbance	6	3
Weight Gain/hyperphagia	6	3
Lethargy	6	3
Abnormal Body Temperature	5	3
Hypertension	5	3
Postoperative Delirium	4	2
Constipation	4	2
Speech difficulties	4	2
Panic attack	4	2
Diarrhea	4	2
Irritability	4	2
Libido decrease/increase	4	2
Increase in drug side effects	4	2
Skin Disorder	4	2
Neuralgia	4	2
Drowsiness	4	2
Palpations Around Neurostimulator	3	2
Neck Pain	3	2
Mania	3	2
Hallucinations	3	2
Palpitations	3	2
Weakness	3	2
Mood swings	3	2
Difficulty voiding/urinary retention	3	2
Back pain	3	2
Electrode revision	3	2
Elective hospitalization	3	2

## Supplementary Materials

The following are available online at https://www.mdpi.com/2077-0383/9/9/2796/s1, Table S1: Complete list of adverse events.
